# A Simple and Rapid Synthesis of Spherical Silver Phosphate (Ag_3_PO_4_) and Its Antimicrobial Activity in Plant Tissue Culture

**DOI:** 10.3390/ijms26157371

**Published:** 2025-07-30

**Authors:** Nongnuch Laohavisuti, Banjong Boonchom, Pesak Rungrojchaipon, Wimonmat Boonmee, Somkiat Seesanong, Sirichet Punthipayanon

**Affiliations:** 1 Department of Animal Production Technology and Fishery, School of Agricultural Technology, King Mongkut’s Institute of Technology Ladkrabang, Bangkok 10520, Thailand; nongnuch.la@kmitl.ac.th; 2 Material Science for Environmental Sustainability Research Unit, School of Science, King Mongkut’s Institute of Technology Ladkrabang, Bangkok 10520, Thailand; 3 Municipal Waste and Wastewater Management Learning Center, School of Science, King Mongkut’s Institute of Technology Ladkrabang, Bangkok 10520, Thailand; 4 Department of Chemistry, School of Science, King Mongkut’s Institute of Technology Ladkrabang, Bangkok 10520, Thailand; pesak.ru@kmitl.ac.th; 5 Department of Biology, School of Science, King Mongkut’s Institute of Technology Ladkrabang, Bangkok 10520, Thailand; wimonmat.bo@kmitl.ac.th; 6 Office of Administrative Interdisciplinary Program on Agricultural Technology, School of Agricultural Technology, King Mongkut’s Institute of Technology Ladkrabang, Bangkok 10520, Thailand; somkiat.se@kmitl.ac.th; 7 Department of Sports Science, Faculty of Physical Education, Srinakharinwirot University, Bangkok 10110, Thailand

**Keywords:** silver phosphate, tissue culture, microorganism, antibacterial activity

## Abstract

A simple and rapid precipitation process was successfully employed to prepare silver phosphate (SP, Ag_3_PO_4_). Two different phosphate sources: diammonium hydrogen phosphate ((NH_4_)_2_HPO_4_) and dipotassium hydrogen phosphate (K_2_HPO_4_) were applied separately as the precursor, obtaining ((NH_4_)_2_HPO_4_)^−^ and K_2_HPO_4_^−^ derived SP powders, named SP-A or SP-P, respectively. Fourier transform infrared (FTIR) spectra pointed out the vibrational characteristics of P–O and O–P–O interactions, confirming the presence of the PO43– functional group for SP. X-ray diffraction (XRD) patterns revealed that the SP crystallized in a cubic crystal structure. Whereas the field emission scanning electron microscope (FESEM) exposed spherical SP particles. The potentially antibacterial activity of SP-A and SP-P against bacterial *Bacillus stratosphericus*, yeast *Meyerozyma guilliermondii*, and fungal *Phanerodontia chrysosporium* was subsequently investigated. All studied microorganisms were recovered and isolated from the aquatic plant during the tissue culture process. The preliminary result of the antimicrobial test revealed that SP-A has higher antimicrobial activity than SP-P. The superior antimicrobial efficiency of SP-A compared to SP-P may be attributed to its purity and crystallite size, which provide a higher surface area and more active sites. In addition, the presence of potassium-related impurities in SP-P could have negatively affected its antimicrobial performance. These findings suggest that SP holds potential as an antimicrobial agent for maintaining sterility in tissue cultures, particularly in aquatic plant systems. The growth of both *B*. *stratosphericus* and *M*. *guilliermondii* was suppressed effectively at 30 ppm SP-A, whereas 10 ppm of SP-A can suppress *P. chrysosporium* development. This present work also highlights the potential of SP at very low concentrations (10–30 ppm) for utilization as an effective antimicrobial agent in tissue culture, compared to a commercial antimicrobial agent, viz., acetic acid, at the same concentration.

## 1. Introduction

Tissue culture, also known as micropropagation, is one of the most effective techniques for the in vitro propagation of numerous valued plant species in a controlled environment [1]. It involves culturing plant cells, tissues, or organs on a nutrient medium under sterile conditions to create multiple clones of the parent plant. Tissue culture is useful for propagating plants that are difficult to propagate through conventional techniques, conserving endangered species, and producing disease-free plants [2]. However, one of the significant challenges in tissue culture is the contamination by various microorganisms, which can severely impact the success rate of plant regeneration [3]. Bacterial and fungal contaminants are among the most common issues, originating from explants, culture media, or equipment [4]. The most frequent microbial contaminants include species from the genera *Pseudomonas*, *Bacillus*, *Escherichia* (bacteria) [5], and *Aspergillus*, *Penicillium*, and *Fusarium* (fungi), which rapidly proliferate in the nutrient-rich environment of culture media [6,7]. These contaminants not only hinder plant growth but also lead to tissue necrosis and the loss of valuable cultures [8], making effective disinfection strategies important to maintaining sterile conditions in preparing explants [9].

Commonly used disinfectants such as sodium hypochlorite (NaOCl) [10], ethanol (CH_3_CH_2_OH) [8], mercury chloride (HgCl_2_) [11], and chlorine dioxide (ClO_2_) [12] are preferred for their broad antimicrobial fields due to their rapid bactericidal action, solubility in water, and relative stability. In cases where fungi cause contamination, specific fungicides such as carboxin (C_12_H_13_NO_2_S) [13], benomyl (C_14_H_18_N_4_O_3_) [14], carbendazim (C_9_H_9_N_3_O_2_) [15], and copper hydroxide (Cu (OH)_2_) [16] are often used. However, these substances are expensive, meaning their use can significantly increase the cost of tissue culture operations. Moreover, no standard decontamination protocol works for all species [17], and no single sterilization method is sufficient for every situation [8]. Contaminants must be eliminated without harming the plant cells; these sterilizing agents are often toxic to plant tissue. Yildiz & Er [18] reported that increasing the concentration of NaOCl solution negatively affected seedling growth and shoot regeneration in flax (Linum usitatissimum). Similarly, in *Lycopersicon esculentum*, sterilization methods influenced the number and size of stomata and cells and the total chlorophyll content [19].

Due to the strong antimicrobial property, silver (Ag)^−^ based materials have been successfully applied to control the microbial contaminants in the processing of plant tissue culture [20,21], such as silver nitrate (AgNO_3_) [22] and silver phosphate (Ag_3_PO_4_) [23]. The mechanism of action involves the release of silver (Ag^+^) ions, which can disrupt microbial cell membranes, interfere with metabolic processes, and ultimately lead to cell death [21]. Additionally, the photocatalytic property of Ag_3_PO_4_ under light exposure produces reactive oxygen species (ROS) such as superoxide (•O_2_^−^) and hydroxyl (•OH) radicals [24], further enhancing its antimicrobial efficiency by causing oxidative damage to microbial cells [25]. This dual mechanism offers significant potential for maintaining aseptic conditions in tissue culture, reducing the reliance on traditional chemical disinfectants, which may pose risks to plant tissues. In addition, phosphorus (P) is an important macronutrient in plant tissue culture, serving as a fundamental component in numerous biochemical and physiological processes that govern in vitro growth and development. As a structural element of nucleic acids (deoxyribonucleic acid (DNA) and ribonucleic acid (RNA)), phospholipids, and energy-rich compounds like adenosine triphosphate (ATP), P plays a pivotal role in sustaining cellular metabolism, energy transfer, and genetic regulation [26]. The continued increase in the production of Ag_3_PO_4_ and its widespread application in agriculture has increased the chances for entry into the soil. However, to the best of our knowledge, there is currently a lack of published studies investigating the direct application of Ag_3_PO_4_ as an antimicrobial agent in plant tissue culture systems. This study aims to fill that gap by demonstrating the synthesis and direct application of Ag_3_PO_4_ as an antimicrobial agent for microbial control during tissue culture procedures.

The synthesis of Ag_3_PO_4_ and its composite has garnered significant interest due to their potential applications in various fields, particularly in agricultural [23], photocatalytic, and biomedical applications [27]. Ag_3_PO_4_ can be synthesized using coprecipitation [28], microwave [29], sonochemical and hydrothermal methods [30], and solvothermal methods [31]. Although these advanced synthetic methods have traditionally been used for nanoparticle synthesis, their drawbacks, including cost, time consumption, and environmental concerns, have led to increased interest in simple and rapid synthesis methods. Thus, this work aims to synthesize Ag_3_PO_4_ using a simple and rapid precipitation method and explore its preliminary antimicrobial application in plant tissue culture. The antimicrobial activity of the synthesized Ag_3_PO_4_ was evaluated for use as a disinfectant in the tissue culture process, specifically against common bacterial and fungal contaminants. The findings obtained in this study on the effectiveness of this material as an antimicrobial agent in plant tissue culture highlight its role in enhancing plant growth and resistance to microbial contaminants.

## 2. Results and Discussion

### 2.1. Physicochemical Characteristics of Ag_3_PO_4_

#### 2.1.1. Functional Group of Ag_3_PO_4_

**Figure 1:**
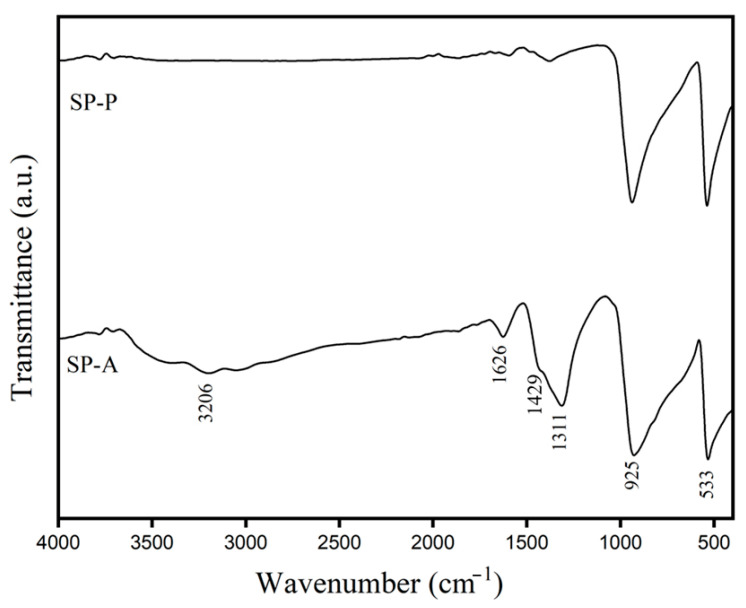
Fourier transform infrared (FTIR) spectra of Ag_3_PO_4_ prepared from (NH_4_)_2_HPO_4_ (SP-A) and from K_2_HPO_4_ (SP-P).

Figure 1 exhibits the FTIR spectra of two different Ag_3_PO_4_ (SP) products that were prepared by using (NH_4_)_2_HPO_4_ (SP-A) and K_2_HPO_4_ (SP-P) as starting materials. The characteristic peaks of the PO_4_^3−^ group in the synthesized Ag_3_PO_4_ are found for both SP-A and SP-P samples. The strong absorption peaks observed at ~925 and ~533 cm^−1^ corresponded to the P–O stretching and O–P–O bending vibrational modes of PO_4_^3−^, respectively [32]. The absorption band at 1429 cm^−1^ corresponded to the P=O stretching of PO_4_^3−^ [33]. The absorption bands at 3206 and 1626 cm^−1^ were observed in the FTIR spectrum of the SP-A sample, compared to that of SP-P. These characteristic absorptions are identified as the O−H stretching and H−O−H bending vibrational modes, respectively, of absorbed water (H2O) on the sample surface [33]. Therefore, the SP-A sample tends to absorb moisture on its surface more than the SP-P. Notably, the strong band with the absorption of 1311 cm^−1^ was clearly observed for the SP-A sample. This band is also attributed to the H−O−H bending vibrational mode of absorbed water; however, this absorption is not the traditional H−O−H bending mode. It could be referred to as the very low H−O−H bending vibration of water [34,35].

#### 2.1.2. Crystal Structure of Ag_3_PO_4_

**Figure 2:**
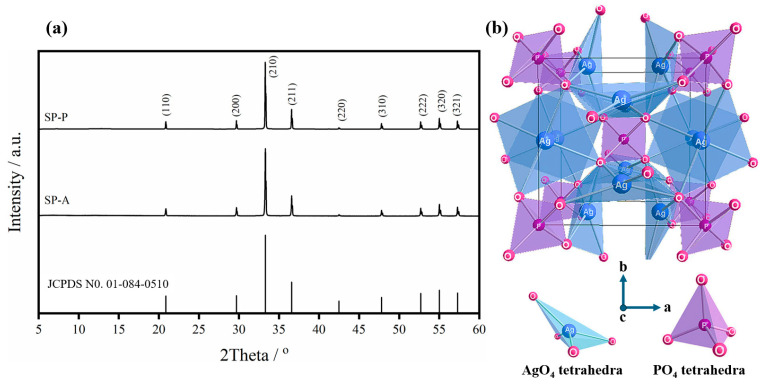
(**a**) X-ray diffraction (XRD) patterns of two Ag_3_PO_4_ products prepared from (NH_4_)_2_HPO_4_ (SP-A) and K_2_HPO_4_ (SP-P), with the standard diffraction file of Ag_3_PO_4_ (JCPDS No. 01-084-0510). (**b**) Cubic crystal structure of Ag_3_PO_4_ consisting of the AgO_4_ and PO_4_ tetrahedra [37].

Figure 2 presents the crystallographic characteristics of the synthesized Ag_3_PO_4_ products (SP-A and SP-P) prepared from different phosphate sources. XRD patterns of both SP-A and SP-P samples, as presented in Figure 2a, are in good agreement with the body-centered cubic (BCC) phase of Ag_3_PO_4_ according to the JCPDS No.01-084-0510 [36]. The space group of cubic Ag_3_PO_4_ is (space group #218), and the lattice parameter of cubic Ag_3_PO_4_ is a = 6.0110 Å, with a lattice angle of α = 90.00°. The cell volume and the number of formula units per unit cell (Z) are 217.15 Å and 2, respectively. Both SP-A and SP-P exhibit intense diffraction peaks (2θ) at ~20.88°, ~29.70°, ~33.30°, ~36.58°, ~42.49°, ~47.80°, ~52.70°, ~55.03°, and ~57.29°, which correspond to the (110), (200), (210), (211), (220), (310), (222), (320), and (321) crystal planes of cubic Ag_3_PO_4_, respectively. In the cubic structure (Figure 2b), Ag and P atoms are each coordinated to four O atoms, producing tetrahedral AgO_4_ and PO_4_ structures. Each O atom connects to three Ag atoms and one P atom. The AgO_4_ tetrahedra are highly distorted within the crystal lattice due to the difference in electronegativity (EN) values between P and Ag [37], leading to the changeability between the three angles of O–Ag–O bonds, viz., 93.54°, 93.61°, and 93.69° [38].

The previous research reported that the precipitation technique can generate a side chemical reaction, which can then generate impurities. Amornpitoksuk et al. [39] precipitated the Ag_3_PO_4_ sample by using sodium orthophosphate (Na_3_PO_4_) as the PO_4_^3−^ precursor, reacting with AgNO_3_ under an aqueous-based reaction. After stirring the yellow suspension at 80 °C for 1 h, the silver oxide (Ag_2_O) was partially mixed with the Ag3PO4 powders. The presence of this impurity could be described through the influence of the acid-base conditions of the reaction solution. The utilized PO_4_^3−^ precursor, such as (NH_4_)_2_HPO_4_ or K_2_HPO_4_, is an acidic salt that can dissolve in water, blocking the generation of AgOH due to the presence of H+ species. However, when basic Na_3_PO_4_ salt dissolves in water, OH^−^ species are produced, generating AgOH and subsequently transforming to Ag_2_O [39,40]. In addition, metallic Ag (Ag0) was also not observed, indicating that the synthetic reaction could not produce a reducing agent during the 3Ag^+^-PO_4_^3−^ nucleation and Ag_3_PO_4_^−^ particle growth processes. As demonstrated in Figure 2a, the well-matched diffraction peaks demonstrated that the synthetic technique employed in this work revealed its potential to precipitate pure Ag_3_PO_4_ crystallites. All observed diffraction peaks were indexed as cubic Ag_3_PO_4_, and other impurities were not observed, such as Ag_2_O and metallic Ag.

The crystallite sizes (*S_c_*) of each synthesized SP-A and SP-P powder were calculated using the first three highest diffraction peaks [(210), (211), (200) planes] and Scherrer’s equation, as demonstrated in Equation (1) [41]. The calculated crystallite sizes of SP-A and SP-P are 115.49 and 135.97 nm, respectively.

(1) $$S_{c}=\frac{0.94\lambda }{\beta \cdot \cos\theta }$$

where *λ* is the employed X-ray wavelength (0.154059 nm), *β* is the full width at the half maximum (FWHM in radians) of each investigated diffraction peak, and *θ* is the diffraction peak angle.

#### 2.1.3. Morphology of Ag_3_PO_4_

**Figure 3:**
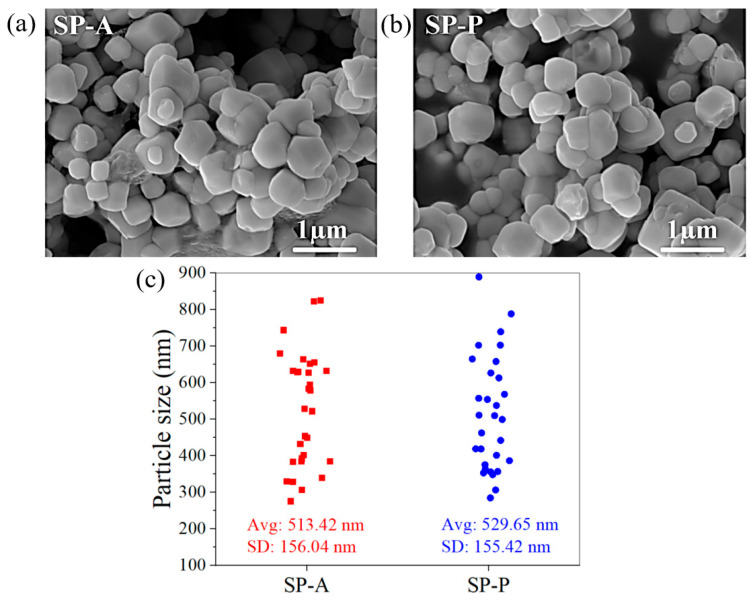
Morphological characteristics of Ag_3_PO_4_ products prepared from (**a**) (NH_4_)_2_HPO_4_ (SP-A) and (**b**) K_2_HPO_4_ (SP-P). (**c**) Particle size distributions (n = 30 particles) of SP-A and SP-P measured by ImageJ software. Data are expressed as mean and standard deviation (SD). The average particle size of SP-A was 513 ± 156 nm, and SP-P was 530 ± 155 nm.

Figure 3a,b show morphologies and particle sizes of Ag_3_PO_4_ (SP-A and SP-P) examined by the FESEM technique. The morphological images of SP-A and SP-P display spherical-shaped crystals. The particle size of materials is an essential parameter that directly influences their application. Therefore, the particle sizes of both SP-A and SP-P samples were determined using ImageJ software [42]. The particle size distributions (n = 30 particles) are presented in Figure 3c. The average particle sizes of SP-A and SP-P are ~513 (±156) nm and ~530 (±155) nm, respectively.

Comparative analysis revealed that the particle size of the synthesized Ag_3_PO_4_ samples, derived from both (NH_4_)_2_HPO_4_ and K_2_HPO_4_, was markedly smaller than the Ag_3_PO_4_ prepared in the literature. Febiyanto et al. [43] precipitated Ag_3_PO_4_ through the reaction between Ag^+^ and PO_4_^3−^ in an aqueous-based solution, and the particle sizes of Ag_3_PO_4_ were in the range of ~0.5–2 µm. Moreover, the utilization of aqueous-based solutions led to the precipitation of unhomogenized particles. Two mixed morphologies between spherical and irregular shapes were obtained. Although the ammonia solution (25%) was added to the reaction solution, the large particle sizes (~0.2–1 µm) were still observed. Therefore, the addition of ethanol into the water in the present work, generating an ethanol–water medium (50% *v*/*v*), resulted in a smaller particle size (~500 ± 155 nm), and the particles were uniform (spherical shape) compared to the Ag_3_PO_4_ precipitated in an aqueous-based solution. It is noted that the crystallite sizes of SP-A and SP-P, calculated from XRD using the Scherrer equation, were smaller than the corresponding particle sizes observed in FESEM images. This difference is expected, as each particle seen under FESEM may be composed of several crystallites aggregated together. Crystallite size represents the size of a single coherent diffraction domain, whereas particle size refers to the whole observable structure, which may consist of multiple crystallites.

#### 2.1.4. Chemical Composition of Ag_3_PO_4_

XRF analysis is an effective technique to determine quantitatively and qualitatively chemical compositions [44] of Ag_3_PO_4_ products prepared from (NH_4_)_2_HPO_4_ (SP-A) and K_2_HPO_4_ (SP-P), and the corresponding results are demonstrated in Table 1. The XRF results revealed that Ag_2_O and P_2_O_5_ are the major chemical components of both SP-A and SP-P, with percentages of more than 98%. Other components with a total percentage less than 2% were also determined from the XRF technique.

Notably, ~1.5% of K_2_O was observed in the case of the SP-P sample, indicating the utilization of K_2_HPO_4_ for the synthesis of Ag_3_PO_4_ due to the co-precipitation of potassium compounds in the synthesis process. As shown in the chemical contents, Ag_3_PO_4_ prepared from (NH_4_)_2_HPO_4_ (SP-A) had high purity (99.9%) of Ag_2_O + P_2_O_5_, with an Ag: PO_4_ weight percentage ratio of 3.43:1. However, due to the presence of K_2_O, SP-P has lower purities (98.3%), with Ag:PO_4_ of 3.06:1. These findings pointed out that (NH_4_)_2_HPO_4_ is an effective source of PO_4_^3−^ for the synthesis of Ag_3_PO_4_ with high purity and Ag content.

### 2.2. Antimicrobial Performance of Ag_3_PO_4_

#### 2.2.1. Isolation and Identification of Contaminants

**Figure 4:**
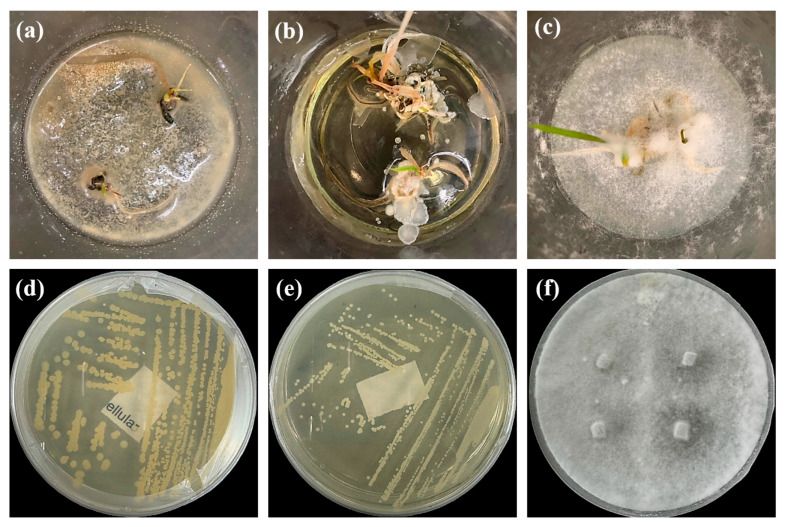
Microbial contamination in tissue culture flasks (**a**–**c**). Various bacteria and fungi grew around the explants in M-S contaminated media during tissue culture. Parts (**d**–**f**) depict bacterial *B. stratosphericus,* yeast *M. guilliermondii,* and fungal *P. chrysosporium*, respectively.

Microorganisms were isolated from contaminated cultures (Figure 4a–c), and pure cultures were obtained. Genomic DNA was extracted and used as a template to amplify the 16S rDNA gene for bacteria and the 18S rRNA gene for fungi, using universal primers [45]. The resulting sequences were analyzed through the National Center for Biotechnology Information–Basic Local Alignment Search Tool (NCBI–BLAST) algorithm for species identification. As presented in Table 2, the contaminants were classified into three taxa, including bacterial *Bacillus stratosphericus,* yeast *Meyerozyma guilliermondii,* and fungal *Phanerodontia chrysosporium*, respectively.

The bacterial *B. stratosphericus* (Figure 4d), an endophytic strain, was identified. This species was previously isolated as an endophyte from the bulbs of *Lilium wardii* [46]. Although endophytic *Bacillus* species generally benefit plants in natural environments, they can cause significant contamination issues during in vitro propagation [47]. Yeast contamination in plant tissue cultures has also been documented. Leifert et al. [48] reported that 78% of yeast strains isolated from contaminated cultures belonged to the Candida genus. In the present work, yeast *M. guilliermondii* (formerly known as *Candida guilliermondii*) was identified. Figure 4e represented a significant portion of the yeast isolates, which constituted 45% of the yeast contaminants. The fungal *P. chrysosporium* (synonym: *Chrysosporium pruinosum*) was also identified (Figure 4f), which aligns with previous reports of fungal contamination in *Musa* spp. tissue cultures [47,49]. These isolated microbial strains were further used to evaluate the antimicrobial efficiency of Ag_3_PO_4_ in a further study.

#### 2.2.2. Antimicrobial Activity Result

The antimicrobial efficiency of the synthesized Ag_3_PO_4_ derived from different phosphate sources was investigated against the obtained bacterial and fungal contaminants in tissue culture, as listed in Table 2. The preliminary result pointed out that Ag_3_PO_4_ prepared from (NH_4_)_2_HPO_4_ (SP-A) showed superior antimicrobial efficiency to that prepared from K_2_HPO_4_ (SP-P). This superior antimicrobial activity of (NH_4_)2HPO_4_^−^-derived Ag_3_PO_4_ (SP-A) could be described through the higher product purity with smaller crystallite size, as presented in Figure 3. The smaller crystallite size of materials commonly relates to the higher surface area with sufficient active sites for biochemical activity. The lower antimicrobial efficiency of SP-P could also be ascribed to the higher impurities, especially K_2_O, as presented in the XRF chemical composition (Table 1). The presence of potassium (K)plays a crucial role in microbial growth and community structure, influencing both the types and activity of microorganisms in various environments. This main point influences the low antimicrobial efficiency of the K_2_HPO_4_ (SP-P) applied. In addition, although Ag_3_PO_4_ is poorly soluble in water, it was first pre-dissolved using a small volume of acetic acid before dilution, allowing better dispersion and bioavailability in the test medium. This approach enabled its effective use in antimicrobial testing, while minimizing solubility-related limitations. However, the potential influence of dissolved Ag^+^ or released phosphate ions on plant nutrient uptake remains a topic for further investigation.

Ag-based antimicrobial agents can release Ag^+^ ions for inhibiting and destroying microbes [21,23]. Therefore, the higher Ag content of SP-A (Table 1) is a significant factor that improved antimicrobial efficiency compared to SP-P. According to the preliminary result, only (NH_4_)_2_HPO_4_^−^-derived Ag_3_PO_4_ (SP-A) was selected to further investigate its antimicrobial activity. The antimicrobial activity result of Ag_3_PO_4_ (SP-A) is presented in Table 3. The results demonstrate that Ag_3_PO_4_ exhibits significant antimicrobial activity against both bacteria (*B. stratosphericus*) and yeast (*M. guilliermondii*), and its antimicrobial activity increased with increasing dosage (Ag_3_PO_4_ concentration).

As demonstrated in Table 3, without the addition of Ag_3_PO_4_ (control condition), the inhibition zones for both bacterial *B. stratosphericus* and yeast *M. guilliermondii* were not observed. This result pointed out the stability of both microorganisms. With the addition of the lowest Ag_3_PO_4_ concentration (5 ppm) studied, the inhibition zones for both studied microorganisms were not observed. This finding suggested that 5 ppm of Ag_3_PO_4_ is not sufficient to inhibit the development of microbes. A similar result was also observed for fungal *P. chrysosporium*. When 5 ppm of Ag_3_PO_4_ was added to the testing system, the colony diameter, compared to a control condition, was not different, confirming the insufficient amount for microbial reduction. However, utilizing Ag_3_PO_4_ at least 10 ppm, antimicrobial efficiency of Ag3PO4 was observed, indicating antimicrobial activity over Ag_3_PO_4_, and fungal *P. chrysosporium* was completely inhibited and destroyed at this Ag_3_PO_4_ concentration (10 ppm).

The antibacterial mechanism of the synthesized Ag_3_PO_4_could be described based on its physicochemical properties. Ag_3_PO_4_ and its released Ag^+^ can interact in both direct and indirect pathways with targets before destroying microbial development [21]. Ag_3_PO_4_ can react with carboxylic (–COOH) and amine (–NH–) groups in microbial cell walls, resulting in the destruction of the cell wall. Ag^+^ can adhere to the cell wall and cell membrane via electrostatic attraction with the sulfhydryl (–SH) group, enhancing the permeability of the cytoplasmic membrane, hence damaging the microbial envelope. Ag_3_PO_4_ and Ag^+^ can alter the environmental pH within microbes, denature ribosomes, and suppress protein synthesis, hence destroying microbial metabolism. Microbials’ DNA can be adhered to Ag_3_PO_4_ and Ag^+^, preventing the replication and cell proliferation of microbials [21]. In addition, Ag_3_PO_4_ is an effective semiconductor, which can generate electron-hole (e^−^-h^+^) pairs under visible light. These Ag_3_PO_4_-derived charge carriers can induce the formation of high oxidative species, including •O_2_^−^ and •OH radicals, which negatively affect the cell membrane, protein, and DNA, preventing microbial development [23].

The antimicrobial results, as presented in Table 3, indicated that the inhibition zone increased with increasing Ag_3_PO_4_ concentrations. The inhibition zone for bacterial *B. stratosphericus* increased from 6.10 ± 0.56 mm at 5 ppm Ag_3_PO_4_ to 20.50 ± 1.91 mm at 30 ppm Ag_3_PO_4_. Similarly, for yeast *M. guilliermondii*, inhibition zones spanned from 11.83 ± 2.70 mm at 5 ppm Ag_3_PO_4_ to 22.33 ± 4.23 mm at 30 ppm Ag_3_PO_4_. Statistically significant differences (*p* < 0.05) were observed between the control and all Ag_3_PO_4_ concentrations 10 ppm for both *B. stratosphericus* and *M. guilliermondii* microbials. The increase in antimicrobial efficiency with increasing Ag_3_PO_4_ concentration, suggesting a dose-dependent response, is consistent with findings reported in the literature. Dânoun et al. [50] observed significant antibacterial effects of Ag_3_PO_4_ against *Escherichia coli* and *Staphylococcus aureus*. The inhibition zone affected by Ag_3_PO_4_ against *E. coli* increased from 10.00 to 13.50 mm when the concentration of Ag_3_PO_4_ increased from 0.125 to 1 mg/mL, respectively. A similar result was observed in the case of *S. aureus* microbial. Ag_3_PO_4_ with 0.125 mg/mL presented the inhibition zone of 9.2 mm, whereas a 12.01 mm inhibition zone was obtained by employing 1 mg/mL Ag_3_PO_4_ [50].

The Ag_3_PO_4_ was also evaluated mainly by inactivating hyphal growth via the poison food method for antifungal activity. Therefore, the diameter of the mycelial colony was measured, and agar plate colony images for fungal *P. chrysosporium* are presented in Table 4. Visually, treating *P. chrysosporium* with increasing concentrations of Ag_3_PO_4_ showed a progressive inhibitory effect. Obviously, complete inhibition was achieved at 10 ppm and above concentrations, while no inhibition was observed at 5 ppm. These findings further confirm that at least 10 ppm is a sufficient concentration of Ag_3_PO_4_ for utilizing for the effective inhibition of the studied microbial system.

For the unidentified fungal species, a gradual decrease in colony diameter was observed with increasing Ag_3_PO_4_ concentrations. The partial inhibition was noted at 5 ppm, with complete inhibition achieved at 20 ppm and above. Over 7 days of incubation in vitro, it was found that Ag_3_PO_4_ restrained the mycelial growth of both fungi tests, displaying dramatic concentration-dependent toxicity effects, which was in accordance with the literature for Ag_3_PO_4_ [51] or Ag_3_PO_4_ [52]. These findings also indicate that Ag_3_PO_4_ is an effective antimicrobial agent against bacterial and fungal contaminants commonly found in tissue culture. The compound demonstrated broad-spectrum activity, inhibiting bacteria, yeast, and filamentous fungi.

Generally, acetic acid is an effective and economical antimicrobial agent, with typical working concentrations ranging from 0.5% to 1% (5000–10,000 ppm) reported in previous studies [53]. However, the results observed in this work pointed out that employing 30 ppm of acetic acid did not inhibit the development of the studied microbials. As presented in Table 4, the use of the same concentration (30 ppm) of two antimicrobial agents, between commercial acetic acid and synthesized Ag_3_PO_4_ (SP-A), only SP-A presented antimicrobial activity against the studied microbes. The absence of antimicrobial activity of acetic acid could be described through the concentration utilized in the process. Fraise et al. [54] investigated the antimicrobial activity of acetic acid and reported that acetic acid could inhibit the development of microbes when the concentration of acetic acid is higher than 1660 ppm. Consequently, Ag_3_PO_4_ synthesized by a simple precipitation method demonstrates promising antimicrobial efficiency against common bacterial and fungal contaminants in tissue culture. Its broad-spectrum activity and effectiveness suggest a potential candidate for use as a disinfectant in tissue culture processes. However, further studies on its effects on plant tissues and long-term exposure would be necessary to evaluate this application fully. Further research is needed to evaluate the long-term effects of SP on plant growth and development. Further investigation into the optimal concentrations for various microbial species could enhance the application of the SP in improving the sterility and quality of plant tissue cultures.

## 3. Materials and Methods

### 3.1. Synthesis and Characterization of Ag_3_PO_4_

#### 3.1.1. Starting Materials

All starting materials employed in this research were AR grade and used without further purification processes. Silver nitrate (AgNO_3_) with a purity of 99%, received from Merck, was utilized as an Ag^+^ source. Diammonium hydrogen phosphate ((NH_4_)_2_HPO_4_) and dipotassium hydrogen phosphate (K_2_HPO_4_) with a purity of 99%, purchased from KemAus, were utilized separately as PO_4_^3−^ sources. The solvent used for dissolving all starting materials in this work was prepared by mixing absolute ethyl alcohol (CH_3_CH_2_OH, 99.9%, AR grade, Q RëC™) and deionized (DI) water (Milli-Q^®^ (Rockville, MD, USA) EQ 7000 ultrapure water system, 18.2 MΩ·cm) at a volume ratio of 1:1. AgNO_3_, (NH_4_)_2_HPO_4_, and K_2_HPO_4_ were dissolved separately in the prepared solvent to obtain 0.20 mol/L solutions for use as precursors in the preparation of Ag_3_PO_4_.

#### 3.1.2. Ag_3_PO_4_ Preparation

Based on the chemical stoichiometry, a mole ratio of Ag^+^ source per PO_4_^3–^ source of 3:1 was utilized for the precipitation of Ag_3_PO_4_ with high purity. In a typical procedure, 100.00 mL of (NH_4_)_2_HPO_4_ solution (0.20 mol/L) was slowly added into a beaker containing 300.0 mL of AgNO_3_ solution (0.20 mol/L) under a continuous stirring process (400 rpm). The mixture, subsequently formed precipitates, was stirred for 30 min to achieve complete reaction. After that, the obtained yellow suspension was filtered through a Whatman filter paper (No.42) and washed with DI water and ethanol each three times. The obtained precipitates were dried at 60 °C until dried Ag_3_PO_4_ powders were completely observed. All synthetic steps were repeated using K_2_HPO_4_ instead of (NH_4_)_2_HPO_4_. The prepared Ag_3_PO_4_ powders obtained from ammonium-based phosphate ((NH_4_)_2_HPO_4_) or potassium-based phosphate (K_2_HPO_4_) precursors were labeled as SP-A or SP-P, respectively. Since silver phosphate (Ag_3_PO_4_) is sparingly soluble in water, a small amount of acetic acid was used to facilitate its dissolution. The prepared compounds were first digested in acetic acid and then diluted with deionized water to prepare a stock solution at a concentration of 100 ppm. This stock was subsequently diluted to obtain working solutions of 5, 10, 15, 20, 25, and 30 ppm for antimicrobial testing.

#### 3.1.3. Characterization of Ag_3_PO_4_

The functional group that existed in the synthesized SP-A and SP-P samples was analyzed by the Fourier transform infrared (FTIR) spectrophotometer (Spectrum GX, Perkin Elmer, Waltham, MA, USA). By employing the KBr pellet technique, the spectral profile was recorded in the wavenumber range from 4000−400 cm^−1^ with 8 scans and a resolution of 4 cm^−1^. The crystal structure and phase purity of the sample were characterized by X-ray diffractometer (XRD, Rigaku-MiniFlex, The Woodlands, TX, USA) with Cu-Kα radiation (λ = 0.15406 nm). The XRD pattern of the sample was analyzed at 2θ angles from 5−60° with an increment of 0.01° under the scan speed of 1 s/step, and electron acceleration at 30 kV and 40 mA. The obtained diffraction was compared with the Joint Committee on Powder Diffraction Standards (JCPDS) database to clarify the crystalline characteristics and phase purity. The surface morphology and elemental composition of the sample were characterized by field emission scanning electron microscope (FESEM, LEO VP1450, Zeiss, Jena, Germany, operating at 15 kV) and X-ray fluorescence (XRF, SRS 3400, Bruker, Billerica, MA, USA), respectively. Prior to FESEM operation, the sample was coated by gold to increase its conductivity with charging effect reduction. Particle size distribution was determined by analyzing field emission scanning electron microscope (FESEM) images using ImageJ (version 1.47) software. The scale was first set using the provided image scale bar. Particle diameters were measured manually using the “Straight Line” tool (BLASTn (BLAST+ v2.13.0)) on well-separated particles (n = 30). The mean and standard deviation were calculated from the measured values.

### 3.2. Antimicrobial Activity of Ag_3_PO_4_

#### 3.2.1. Isolation of Contaminating Microorganisms from Aquatic Plant Tissue Culture

Based on phenotypic observation, bacterial contaminants frequently appeared in the Murashige and Skoog (MS) medium within a month [55]. To culture these bacteria, inoculation loops were used to streak them onto nutrient agar (NA) plates (HiMedia^®^, Mumbai, India), followed by incubation at 26 °C for 48 h. Single bacterial colonies were then selected and re-streaked on fresh NA plates. Once the morphology of all colonies on the plates was uniform, a pure bacterial culture was obtained. Similarly, fungal contaminants were also observed in the MS medium. Based on phenotypic characteristics, typical fungi were selected and inoculated onto potato dextrose agar (PDA) plates (HiMedia^®^, Mumbai, India) and incubated at 26 °C for 72 h [56]. After several rounds of subculturing, pure fungal cultures were obtained and stored on PDA plates at 4 °C for further use.

#### 3.2.2. Molecular Microbial Identification

Bacterial cells were washed twice with sterile phosphate-buffered saline (PBS) with a pH of 7.4 [57], and genomic DNA was extracted using the MagPurix^®^ Bacterial DNA Extraction Kit (ZINEXTS, New Taipei City, Taiwan) according to the manufacturer’s protocol. The DNA was stored at −20 °C until utilized and served as a template for the polymerase chain reaction (PCR). The PCR mixture contained 10.0 μL of 2× Taq PCR Master Mix, 0.4 μL of 10 μM forward primer (27F), 0.4 μL of reverse primer (1492R), and 1.0 μL of 500 ng template DNA [58,59]. The final volume was adjusted to 20 μL by the addition of sterile distilled water. PCR amplification was performed on all isolates to target the 16S rDNA gene using universal primers 27F (5′- AGA GTT TGA TCM TGG CTC AG -3′) and 1492R (5′- TAC GGY TAC CTT GTT ACG ACT T -3′). The amplification was carried out in a thermal cycler (BioRad, Hercules, CA, USA) with an initial denaturation at 95 °C for 5 min, followed by 34 cycles of 94 °C for 30 s, 55 °C for 30 s, and 72 °C for 1 min, with a final extension at 72 °C for 7 min [60]. PCR products were analyzed by 1.2% (*w*/*v*) agarose gel electrophoresis (containing GelRed) in 1% tris-acetate-EDTA (TAE) buffer. Gels were visualized and photographed under UV illumination. The Sanger sequencing of the PCR products was analyzed by Macrogen Inc. (Seoul, Republic of Korea). The 16S rDNA sequences were submitted to the NCBI GenBank database and analyzed using the BLASTn algorithm (BLAST+ 2.13.0) [61]. Bacterial species identification was successful, with more than 97% nucleotide identity for the 16S rDNA sequence.

For fungal samples, the hyphae were collected and ground with sterile glass beads, and genomic DNA was extracted using the cetyltrimethylammonium bromide (CTAB) method [62]. This DNA was used as a template for PCR amplification of the 18S ribosomal RNA (18S rRNA) gene, using primers NS1 (5′- GTA GTC ATA TGC TTG TCT C -3′) and NS24 (5′- AAA CCT TGT TAC GAC TTT TA -3′), following a similar protocol as described above.

#### 3.2.3. Antimicrobial Activity Test

The antibacterial activity of Ag_3_PO_4_ against the isolated bacterial contaminants was assessed using the disk diffusion method [63]. Bacterial strains were spread on NA media using a sterile cotton swab. Sterile 6 mm diameter antimicrobial assay disks (Whatman^®^, Piscataway, NJ, USA) were used for the test. The disks were loaded with 10 μL of Ag_3_PO_4_ at 5 to 30 ppm (parts per million) concentrations (aqueous-based solution). The disks were placed on the agar plates and incubated at 37 °C for 24 h [64]. The zone of inhibition was measured after the incubation period [23].

Antifungal activity was evaluated using the poisoned food technique [65]. PDA medium (20 mL) containing different concentrations of Ag_3_PO_4_ was poured into sterilized Petri dishes. The concentrations of Ag_3_PO_4_ used were 5, 10, 15, 20, 25, and 30 ppm. The control condition was also conducted without the addition of Ag_3_PO_4_ (0 ppm). Spore suspensions of the tested fungi were inoculated onto control and poisoned plates using a sterile inoculation needle. The plates were incubated for 7 days at room temperature [66]. The antifungal activity of Ag_3_PO_4_ was assessed based on the inhibition of mycelial growth of the tested fungi.

All experimental data are presented as mean ± standard deviation (SD) based on six replicates (n = 6). Statistical analysis was performed using analysis of variance (ANOVA), followed by Duncan’s new multiple range test (Duncan’s MRT) to determine significant differences among treatments at a confidence level of *p* ≤ 0.05 using the IBM SPSS Statistics version 22.

## 4. Conclusions

An effective antimicrobial Ag_3_PO_4_ (SP) material was successfully synthesized through a rapid and simple precipitation technique. Employing two different phosphate (PO_4_^3−^) sources: (NH_4_)_2_HPO_4_ and K_2_HPO_4_, SP-A and SP-P were obtained, respectively. The FTIR spectral result shows the vibrational characteristics of the PO_4_^3−^ functional group, pointing out the characteristic of Ag_3_PO_4_. XRD patterns revealed that Ag_3_PO_4_ crystallizes in a cubic crystal structure with crystallite sizes of 115.49 and 135.97 nm for SP-A and SP-P, respectively. The spherical particles, imaged by the FESEM technique, were also observed for both SP-A and SP-P samples; however, Ag_3_PO_4_ prepared from (NH_4_)_2_HPO_4_ (SP-A) has a particle size (~513 nm) with more purity compared to SP-P (~530 nm). XRF analysis further confirmed the higher phase purity of SP-A (99.9%) compared to SP-P (98.3%), with the presence of potassium (1.5%), which plays a crucial role in microbial growth and community structure, influencing both the types and activity of microorganisms in various environments. The smaller crystallite size and higher purity of SP-A result in synergistically enhancing its antimicrobial performance. The antimicrobial activity of SP-A was evaluated against three different microbial species, including bacterial *B. stratosphericus*, yeast *M. guilliermondii*, and fungal *P. chrysosporium*. The antimicrobial result indicated that SP-A presented its effective antimicrobial efficiency, and the inhibition of microbial growth increased with increasing SP-A concentration. SP-A also presented excellent efficiency at low concentration compared to commercial antimicrobial agents such as acetic acid for all microbials studied. However, further studies, particularly those evaluating phytotoxicity and plant regeneration, are required before its application in live plant systems can be fully validated.

## Figures and Tables

**Table 1:** Chemical compositions of Ag_3_PO_4_ powders prepared from (NH_4_)_2_HPO_4_ (SP-A) and K_2_HPO_4_ (SP-P).

Chemical Compositions			Chemical Contents/wt%	
			SP-A	SP-P
Major components	Silver oxide	Ag_2_O	87.2	84.5
	Phosphorus pentoxide	P_2_O_5_	12.7	13.8
Minor components	Silicon dioxide	SiO_2_	0.0250	0.0275
	Sulfur trioxide	SO_3_	0.6900	0.0596
	Potassium oxide	K_2_O	–	1.5100
	Palladium oxide	PdO	0.0261	0.0871
Total purities (Ag_2_O + P_2_O_5_)			99.9	98.3

**Table 2:** The species analysis of microbial contaminants from the contaminated media and the search results from the BLAST analysis of the amplified sequences.

Isolate No.	Species Recognized on BLAST (Best Hit)	GenBank Accession	Identities		Note
			Match/Total	Pct. (%)	
1	*B. stratosphericus*	NR_042336	1496/1498	99.86	Bacteria
2	*M. guilliermondii*	KX258468	1658/1658	100.00	Yeast
3	*P. chrysosporium*	MH047187	1654/1655	99.93	Fungi

**Table 3:** In vitro antimicrobial activity of Ag_3_PO_4_ (SP-A) against isolated microbial contaminants by disk diffusion and poisoned food methods. Data are expressed as mean ± SD (n = 6). Different letters within a column are significantly different according to Tukey’s multiple range test (*p* < 0.05).

Treatments		Zone of Inhibition (mm)		Colony Diameter (mm)
		Bacterial *B. stratosphericus*	Yeast *M. guilliermondii*	Fungal *P. chrysosporium*
Control (DI water)		0.0 ± 0.0 ^a^	0.0 ± 0.0 ^a^	84.60 ± 0.0 ^b^
Ag_3_PO_4_ (ppm)	5	0.0 ± 0.0 ^a^	0.0 ± 0.0 ^a^	84.60 ± 0.0 ^b^
	10	6.10 ± 0.56 ^b^	11.83 ± 2.70 ^b^	0.0 ± 0.0 ^a^
	15	12.06 ± 1.58 ^c^	14.38 ± 0.80 ^bc^	0.0 ± 0.0 ^a^
	20	16.61 ± 0.26 ^d^	15.75 ± 0.49 ^c^	0.0 ± 0.0 ^a^
	25	17.41 ± 0.54 ^d^	16.70 ± 2.25 ^c^	0.0 ± 0.0 ^a^
	30	20.50 ± 1.91 ^e^	22.33 ± 4.23 ^d^	0.0 ± 0.0 ^a^

**Table 4:** Antimicrobial efficiency of Ag_3_PO_4_ (SP-A) against the growth of isolated microbial contaminants (*B. stratosphericus, M. guilliermondii, P. chrysosporium*) using disk diffusion and poisoned food methods under in vitro conditions.

Microbials	[Acetic Acid] 30 ppm	[Ag_3_PO_4_]		
		10 ppm	20 ppm	30 ppm
Bacterial *B. stratosphericus*	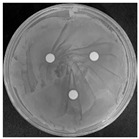	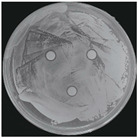	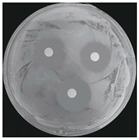	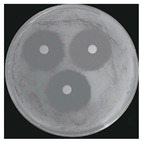
Yeast *M. guilliermondii*	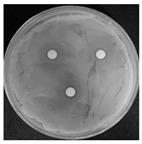	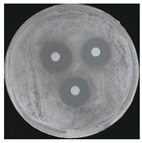	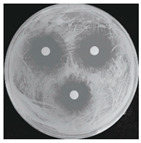	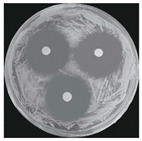
	**[Acetic Acid]** **30 ppm**	**[Ag_3_PO_4_]**		
		**5 ppm**	**10 ppm**	**15 ppm**
Fungal *P. chrysosporium*	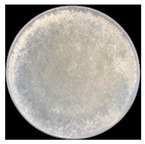	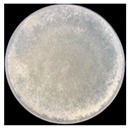	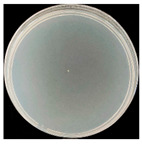	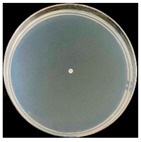

## Data Availability

Data will be made available on request.
